# Too much, too little, or just right? The inverted-U relationship between human-AI collaboration modes and eudaimonic well-being

**DOI:** 10.3389/fpsyg.2026.1747252

**Published:** 2026-09-28

**Authors:** Zhigang Weng, Yu Mi, Xiaoxiao Chen, Qin Li

**Affiliations:** 1 School of Business Administration, Southwestern University of Finance and Economics, Chengdu, China; 2 School of Tourism and Cultural Industries, Sichuan Tourism University, Chengdu, China; 3 School of Finance and Accounting, Chengdu Jincheng College, Chengdu, China

**Keywords:** AI dependence, artificial intelligence, eudaimonic well-being, human-AI collaboration, perceived autonomy

## Abstract

As human-AI collaboration becomes more pervasive, optimizing the distribution of decision-making authority between humans and AI has become a fundamental concern in human-centered AI design. The impacts of different human-AI collaboration modes (human-dominant, human-AI collaboration, and AI-dominant) on users’ eudaimonic well-being, as well as the underlying psychological transmission mechanisms, remain poorly understood. Grounded in self-determination theory, we propose a serial mediation framework linking human-AI collaboration modes to eudaimonic well-being via the sequential mechanisms of AI dependence and perceived autonomy. This framework is rigorously investigated through two scenario-based experiments in the domains of autonomous driving and online shopping, with a particular focus on the differential effects of collaboration modes and the underlying psychological transmission chain. The results reveal that human-AI collaboration mode exhibits an inverted U-shaped relationship with eudaimonic well-being; the level of individuals’ eudaimonic well-being under the human-AI collaboration mode is significantly higher than that under the human-dominant and AI-dominant modes. Additionally, AI dependence and perceived autonomy play a significant serial mediating role in the above relationship. This study constructs and validates the psychological transmission mechanism of how human-AI collaboration affects deep-level well-being, expands the application scope of self-determination theory in human-AI collaboration scenarios, and provides optimization guidelines for AI product design and user experience management. These findings contribute to the design of human-centered AI systems that simultaneously promote technological advancement and human well-being.

## 1 Introduction

With the rapid development of algorithms and generative artificial intelligence (AI), intelligent systems have gradually evolved from auxiliary tools into collaborative cognitive agents. They are deeply involved in human daily judgment, behavior planning, and decision-making (Nambisan et al., 2017; Topol, 2019). AI systems greatly improve task efficiency, optimize resource allocation, and support innovation in industrial, medical, and educational fields (Azeez et al., 2023; Bahoo et al., 2023; Ding et al., 2025). Meanwhile, this deep integration requires in-depth investigation. These issues include consumer well-being changes, excessive user reliance on AI, reduced proactive cognitive ability, and loss of perceived autonomy in algorithm-driven environments (Jiao and Cao, 2024; Peng et al., 2026).

The growing involvement-and in some cases, dominance-of AI in decision-making processes is often attributed to its capacity to process vast amounts of data, recognize complex patterns, and generate timely recommendations. However, the mere combination of human judgment and AI does not automatically yield superior outcomes. A systematic review and meta-analysis revealed that human-AI collaboration does not consistently outperform the better-performing member of the human-AI dyad (Vaccaro et al., 2024). Consequently, complementarity should not be regarded as an inherent property of human-AI interaction, but rather as an achievement that is realized only under certain conditions. Effective collaboration hinges on whether users understand the relative strengths and limitations of both humans and AI, calibrate their reliance on AI appropriately, and retain the capacity to determine when to accept or override AI-generated advice (Hemmer et al., 2025; Steyvers and Kumar, 2024).

Eudaimonic well-being (EWB) originates from Aristotle’s conception of happiness. Existing research on artificial intelligence (AI) and human well-being has primarily focused on AI trust formation, technology acceptance, and user satisfaction (Afroogh et al., 2024; Li et al., 2024; Pham et al., 2024; Weng et al., 2026). However, existing studies pay limited attention to how AI shapes human subjectivity, perceived autonomy, and the construction of personal meaning. Previous studies divide consumer Well-Being into three dimensions: utilitarian well-being, hedonic well-being, and eudaimonic well-being (Chen et al., 2023; Chrisinger et al., 2019). Different from superficial hedonic pleasure and immediate situational satisfaction, eudaimonic well-being refers to a high-order and stable psychological state in which individuals adhere to their authentic self, tap into their inner potential, and achieve comprehensive positive life development. It underpins the release of individual potential through authentic self-expression and continuous growth, representing an in-depth core psychological Well-Being with long-term value (Iqbal and Mansell, 2021; Oh et al., 2024).

According to self-determination theory, individuals’ long-term psychological Well-Being fundamentally depends on the satisfaction of three basic psychological needs: autonomy, competence, and relatedness (Ryan and Deci, 2000). This study focuses on autonomy in human-AI collaboration. At its core, human-AI collaboration involves the allocation and transfer of decision-making control. Unlike traditional interpersonal or task-based settings where all three needs are jointly implicated, AI interaction poses a unique and existential threat specifically to autonomy–as users increasingly succumb to automation bias and algorithmic reliance, their independent judgment is systematically ceded to machine intelligence. This makes autonomy the most theoretically vulnerable and practically consequential need in the AI context. We contend that perceived competence in human-AI collaboration is primarily determined by the AI’s technical performance-such as algorithmic accuracy, response latency, and output quality–rather than by the collaborative structure *per se*. Since our study focuses on the structural configuration of decision authority (i.e., collaboration modes), rather than on AI performance variations, competence is largely held constant across experimental conditions and thus does not serve as a meaningful mediator in our framework. And we do not assume that AI embodies genuine emotional agency; consequently, perceived relatedness in human-AI interaction is more likely to stem from anthropomorphic design features (e.g., voice, avatar, or social cues) than from the mode of collaboration.

In human-AI collaboration scenarios, users’ perceived autonomy is greatly affected by the feedback structure, control logic and explanation mechanism of AI systems (Frank et al., 2026; Oh et al., 2025; Zhao et al., 2025). Most mainstream AI system designs prioritize efficiency and accuracy, but ignore users’ psychological involvement and sense of control during collaboration. This may weaken users’ decision-making initiative and psychological satisfaction (Hsu and Lin, 2023; Saleh, 2025).

Scholars hold inconsistent opinions on the relationship between human-AI collaboration and perceived autonomy. Some studies suggest that appropriate AI support can help users allocate cognitive resources more rationally and enhance individual subjective agency (Leng et al., 2026; Zhang and Tao, 2021; Zhou et al., 2024). Other scholars argue that algorithm intervention inevitably limits the scope of human independent decision-making (Dietvorst et al., 2015; Muhammad et al., 2021; Parasuraman et al., 2000; Pham, 2025). At present, it remains unclear how three typical modes of human-AI collaboration (human-dominant, human-AI collaboration, and AI-dominant) influence individual subjective perception and eudaimonic well-being.

This tension is further illuminated by research on trust and dependence. “Appropriate dependence” refers to the user’s ability to employ an automated system when its capabilities adequately warrant such dependence, while simultaneously recognizing its limitations and intervening when necessary (Lee and See, 2004; Parasuraman and Riley, 1997). Both under-dependence and over-dependence can be problematic: under-dependence prevents users from benefiting from effective technological support, whereas over-dependence may lead them to accept erroneous recommendations or cede judgment prematurely (Klingbeil et al., 2024). In the context of this study, AI dependence is conceptualized specifically as functional dependence on AI within task settings, as opposed to compulsive usage, technological addiction, or loss of behavioral control. Such dependence can be empowering when AI extends users’ capabilities while humans retain ultimate authority over goals, values, and final decisions.

Three limitations persist in the existing literature. First, few studies have compared human-dominated, human-AI collaborative, and AI-dominated modes as ordered configurations of decision-making authority. Second, while prior research has primarily focused on performance, trust, and technology acceptance, comparatively less attention has been paid to eudaimonic well-being. Third, the psychological pathway from collaborative structures to eudaimonic well-being remains poorly understood; in particular, the sequential roles of functional AI dependence and perceived autonomy have yet to be systematically examined.

To address these gaps, this paper constructs a sequential mediation model of human-AI collaboration mode-AI dependence-perceived autonomy-eudaimonic well-being. Two scenario-based experiments are conducted. By comparing three distinct types of human-AI collaboration modes, this study reveals the underlying sequential mechanism of human-AI collaboration on individuals’ value realization. It also extends prior work by the proposition of “appropriate reliance” as the principle of human-AI coexistence (Parasuraman and Riley, 1997). Taking eudaimonic well-being as the dependent variable, this research also responds to the single goal orientation of efficiency-centered design prevailing in current AI systems.

## 2 Theoretical background and research hypotheses

### 2.1 Human-AI collaboration mode and eudaimonic well-being

Human-AI collaboration refers to a new cooperative relationship between humans and AI systems. Both sides exert their complementary strengths, conduct information exchange and coordinate control rights when necessary, and pursue common objectives. This partnership does not require strong interdependence or shared accountability (Kamar, 2016; Vats et al., 2024).

In recent years, scholarly research has laid important theoretical foundations for the study of human-AI collaboration. Puerta-Beldarrain et al. (2025) propose a multi-level integrative framework for human-AI collaboration; Hemmer et al. (2025) theorize complementarity to explain how such collaboration surpasses individual performance; Edwards et al. (2025) adopt a socially shared regulation lens to examine AI-facilitated group coordination; Romeo and Conti (2026) scrutinize automation bias and its adverse effects; Raman et al. (2026) explore the role of digital twins and metaverse technologies in empowering supply chains through human-AI synergy.

Based on the degree of AI involvement in cooperation, human-AI collaboration is divided into three classic modes: human-dominant mode, human-AI collaboration mode, and AI-dominant mode. The three modes reflect the development path of artificial intelligence. AI has gradually evolved from a peripheral auxiliary tool into a core decision-making participant (Sharma et al., 2023; Steyvers and Kumar, 2024). The human-AI collaboration scenario constructed in this study emphasizes AI’s enabling role for individuals. It aims to help users complete tasks efficiently and achieve personal value.

The human-dominant mode preserves individuals’ sense of control; nevertheless, high cognitive load and the constraints of personal competence boundaries restrict eudaimonic well-being, especially in scenarios requiring rapid responses and complex judgment. Excessively high cognitive load not only undermines system efficiency but also consumes personal psychological resources, reducing individuals’ sense of gain and Well-Being (Hou et al., 2024; Nazareno and Schiff, 2021; Sweller, 1988).

The AI-dominant mode improves task efficiency yet may trigger self-esteem threat and algorithm aversion, thereby lowering eudaimonic well-being. When individuals’ behavioral decisions are excessively governed by external systems, their basic psychological needs (autonomy, competence, relatedness) cannot be satisfied. Such heteronomous behavior gradually weakens individuals’ psychological integration and sense of meaning in the long run, and ultimately impairs eudaimonic well-being (Nadarzynski et al., 2019; Novrouzai, 2025; Ryan et al., 2008). Users who fully rely on AI for emotional support tend to experience degraded real-world social competence, which further undermines the social connectedness dimension of eudaimonic well-being (Nayyar and Maqsood, 2026).

Compared with human-dominant and AI-dominant forms of human-AI collaboration, the human-AI collaboration mode adopts a framework in which AI undertakes procedural tasks while humans retain dominance over high-level decision-making. When AI takes charge of supportive tasks and humans maintain decision-making leadership, individuals exhibit a stronger sense of control, as well as higher self-efficacy and cognitive satisfaction. This indicates that the collaborative mode is more conducive to facilitating self-expansion and positive psychological integration, and contributes to sustained, in-depth, self-actualization-oriented Well-Being experiences (Sharma, 2024; Sharma et al., 2023).

Human-AI collaboration mode advantage follows from the joint logic of cognitive load, calibrated trust in automation, Optimal distinctiveness theory and human-AI complementarity. Optimal distinctiveness theory holds that AI-generated solutions are perceived as less unique than human-provided ones, inducing negative consumer responses (Leonardelli et al., 2010). Trust calibrates the divide between empowering and addictive dependence-calibrated trust enables rational use, whereas unexamined trust fosters addictive reliance; perceived autonomy further anchors trust calibration. Cognitive load theory suggests AI reduces load via optimized presentation, yet may incur cognitive debt over time; moreover, AI explanations increase perceived task complexity and can impair user perceptions, depending on cognitive capacity (Sweller, 1988). Human-dominant arrangements preserve authority but leave the user carrying the full information-processing burden. AI-dominant arrangements reduce workload but can induce automation bias, skill displacement, and loss of agency. A collaborative allocation can combine AI’s information-processing capability with human contextual and value judgment. Thus, the middle condition is expected to maximize eudaimonic well-being when it reduces avoidable load without transferring final value-laden control to the system (Lyell et al., 2018).

Therefore, we propose the following hypothesis:

***Hypothesis 1 (H1)***: Different human-AI collaboration modes exert significant differential effects on eudaimonic well-being, presenting an inverted U-shaped relationship. Eudaimonic well-being is highest in the human-AI collaborative mode and lower in both the human-dominant and AI-dominant modes.

### 2.2 Human-AI collaboration modes and AI dependence

The impact of AI technology on eudaimonic well-being does not occur directly but unfolds through a series of internal psychological mechanisms (Filippelli et al., 2026; Gao et al., 2025; Lu et al., 2024; Wang et al., 2025). According to human-AI collaboration theory, users develop varying degrees of cognitive dependence and behavioral compliance as long as AI is involved in task assistance (Bauer et al., 2025; Frenkenberg and Hochman, 2025; Klingbeil et al., 2024).

From the perspective of interpersonal relationship theory, a competence complementary structure exists between humans and AI. AI undertakes procedural tasks such as data processing, while humans are responsible for value judgment and decision-making. Such complementary relationships naturally give rise to dependence (Hancock et al., 2023; Ryan, 2020).

Meanwhile, social exchange theory suggests that the asymmetric distribution of resources such as information and computing power between humans and AI provides the intrinsic motivation for the formation of dependence (Ahmad et al., 2023; Bayor et al., 2025; Davlembayeva et al., 2020; Huang et al., 2023; Jiang, 2010). Accordingly, AI dependence stably exists across the human-dominant, human-AI collaboration, and AI-dominant modes, differing only in magnitude and type. The three modes examined in this study all represent collaborative forms involving AI participation, with distinctions merely in the degree of AI involvement and the allocation of rights and responsibilities.

Artificial intelligence dependence refers to the extent to which users cognitively and behaviorally rely on AI systems (Klingbeil et al., 2024; Zhai et al., 2024). Prior research distinguishes between two primary forms of dependence: addiction-based dependence and empowerment-based dependence (Jia, 2025; Lee et al., 2025). Addictive dependence refers to a strong reliance and craving for a certain substance or behavior, accompanied by a loss of voluntary control. Such dependence exerts significant negative impacts on individuals’ physical and mental health, social functioning, and quality of life (Hunt et al., 2024; Shimu et al., 2024).

Empowering dependence denotes a form of reliance formed by individuals or teams within organizational and technological environments after acquiring additional capabilities, resources, or authority. Unlike the traditional negative conception of dependence, this type of reliance enables individuals and teams to complete tasks more efficiently and improve performance after being empowered. To sustain such high-efficiency functioning, they rely to a certain extent on the support and resources provided by the empowering party (Denecke et al., 2019). The human-dominant mode preserves user control but triggers high cognitive load constrained by individual competence, which discourages users’ beneficial dependence on AI (Li, 2026; Wu et al., 2025). Task difficulty is a critical predictor of cognitive load; higher task difficulty consumes more psychological resources and elevates cognitive load, impairing individual psychological functioning and Well-Being (Darling-Hammond et al., 2020; Ekin et al., 2025; Howard et al., 2020; Pyke et al., 2025; Yousef et al., 2025). In contrast, the extreme AI-dominant mode causes algorithm-induced self-identity threat and value substitution perception, leading to user psychological alienation, trust loss, and reduced beneficial dependence willingness (Bhardwaj et al., 2025). Fundamentally, individuals develop AI dependence via competence complementarity and resource exchange in AI-assisted tasks (Parasuraman and Riley, 1997). Differing from the above two modes, the human-AI collaboration mode frames AI as a human capability extender rather than a substitute. Its core lies in knowledge symbiosis: AI empowers cognitive amplification, while humans undertake core meaning interpretation and value judgment (Zhang et al., 2025).

This empowering dependence lies exactly in the optimal spectrum of dependence: AI undertakes basic algorithmic tasks to free up human cognitive resources, whereas humans retain decision-making leadership and sustain the continuity of meaning perception and perceived control.

Therefore, we propose the following hypotheses:

***Hypothesis 2 (H2)***: There are significant differences in AI dependence across human-AI collaboration modes. AI dependence is highest under the human-AI collaboration mode and lower under both the human-dominant and AI-dominant modes, forming an inverted U-shaped relationship.

### 2.3 AI dependence and perceived autonomy

Self-determination theory (SDT) focuses on individuals’ capacity for agentic self-regulation and posits that autonomy, competence, and relatedness are the three fundamental psychological needs. Among these, autonomy is regarded as the core factor enabling intrinsic motivation and Well-Being (Deci et al., 2017). Autonomy reflects “people’s efforts to be agents of their own actions-to experience themselves as the origin of their behavior” (Williams et al., 1998).

In the era of pervasive AI, perceived autonomy has emerged as a key indicator of collaboration quality between humans and intelligent systems. Unlike traditional human-environment collaboration, autonomy in AI contexts extends beyond mere freedom of choice to encompass whether humans and AI can achieve a state of negotiated autonomy (Laitinen and Sahlgren, 2021; Niu et al., 2024; Shin, 2021).

Empowering AI dependence enhances individuals’ sense of control over tasks through competence complementarity, thereby strengthening perceived autonomy (Yang et al., 2025). From the logic of competence complementarity, AI exhibits advantages in basic tasks such as data processing and pattern recognition. Moderate reliance on AI allows individuals to be relieved from low-value labor and focus on high-order decision-making. Retaining dominance over high-order decision-making makes individuals perceive that their behaviors are under self-control (Li, 2026; Pan et al., 2025).

In this study, the empowering effect is not determined by the quantitative degree of AI involvement, but by the qualitative attributes of human-AI collaboration. All three human-AI collaboration modes involve AI participation and competence complementarity. In real-world scenarios where AI is deeply embedded in decision-making, human-AI collaboration inherently possesses the foundation of empowerment (Lee and See, 2004).

The core qualitative feature of empowerment is reflected in the following arrangement: AI undertakes procedural tasks including data processing and pattern recognition, while humans retain dominance over value judgment and high-order decision-making. Such allocation of responsibilities and authority determines whether the collaboration can form empowering AI dependence, promote perceived autonomy, and ultimately influence eudaimonic well-being, rather than simply depending on variations in the level of AI involvement (Parasuraman and Riley, 1997).

In summary, empowering AI dependence can reinforce autonomy, and there is a positive linear correlation between AI dependence and perceived autonomy.

Therefore, we propose the following hypothesis:

***Hypothesis 3 (H3)***: AI dependence positively predicts users’ perceived autonomy.

### 2.4 Perceived autonomy and eudaimonic well-being

Eudaimonic well-being (EWB) emphasizes the psychological fulfillment derived from realizing one’s potential, pursuing meaningful goals, and constructing personal meaning (Waterman, 1993). Empirical evidence demonstrates that higher levels of perceived autonomy significantly enhance individuals’ sense of meaning and psychological health (Sheldon et al., 2004). For instance, in educational contexts, learners who maintain autonomy in their learning decisions exhibit stronger intrinsic motivation and higher learning Well-Being (Jeno et al., 2019). In organizational settings, perceived autonomy is positively associated with employees’ creativity, job satisfaction, and long-term mental health (Gagné and Deci, 2005). Studies conducted in AI contexts further validate this relationship. When users experience control and freedom of choice during AI collaborations, their sense of meaning and Well-Being increases significantly (Shin, 2021). Thus, perceived autonomy can be viewed as a critical psychological mechanism that predicts positive outcomes in work, learning, and consumption domains (Deci et al., 2017).

***Hypothesis 4 (H4)***: Consumers’ perceived autonomy has a significant positive effect on their eudaimonic well-being.

***Hypothesis 5 (H5)***: AI dependence and perceived autonomy jointly mediate the relationship between human-AI collaboration mode and eudaimonic well-being. Specifically, human-AI collaboration mode influences AI dependence, which in turn affects perceived autonomy, ultimately shaping consumers’ eudaimonic well-being.

In summary, this study develops a theoretical model linking human-AI collaboration modes to consumers’ eudaimonic well-being through the chained mediating effects of AI dependence and perceived autonomy.

## 3 Research design and data analysis

This study adopts a multi-stage experimental research paradigm to sequentially validate the effectiveness of the experimental manipulations, examine the causal effects of human-AI collaboration modes on eudaimonic well-being, and elucidate the underlying psychological mechanisms.

First, a card-sorting pre-experiment is conducted to objectively verify the cognitive discriminability and real-world ecological validity of the three human-AI collaboration modes.

Second, two formal between-subjects experiments are implemented sequentially. Study 1 specifically examines the main effect of human-AI collaboration modes on users’ eudaimonic well-being, and quantitatively tests whether their relationship conforms to the standard inverted U-shaped nonlinear pattern. Based on the validated main effect findings, Study 2 further introduces two continuous mediating variables-AI dependence and perceived autonomy–to verify the sequential mediating path linking human-AI collaboration modes to eudaimonic well-being.

The choice of laptop purchasing decisions and autonomous driving as experimental scenarios was motivated by their distinct yet complementary characteristics. Laptop purchasing entails high information load, readily comparable product attributes, frequent AI-generated recommendations, and reversible decision outcomes. In contrast, autonomous driving represents a state-of-the-art AI application, characterized by high stakes, irreversibility, and strong identity relevance. The two scenarios thus offer complementary contextual variations, enabling a robust examination of human-AI collaboration across divergent risk and reversibility profiles.

Participants were recruited through Credamo, one of the largest online behavioral research platforms in China and a widely adopted participant recruitment platform in behavioral and consumer research. To ensure that participants were familiar with contemporary AI technologies, recruitment was restricted to adults aged between 21 and 40 years who regularly used intelligent digital systems. Before participation, all respondents provided informed consent, and each participant received a small monetary incentive after completing the experiment.

### 3.1 Card-sorting validation

Ten participants were randomly presented with eight shopping scenarios of human-AI collaboration featuring different levels of autonomy (see Supplementary Appendix). Participants were asked to freely categorize the scenarios according to the perceived similarity of decision-right allocation, label each category, and provide explanatory reasons. The results revealed that all participants consistently formed a stable three-category structure, and their descriptions of category characteristics closely corresponded to the predefined dimensions: human-dominant, human-AI collaboration, and AI-dominant.

### 3.2 Experiment 1

This study aims to test Hypothesis 1, which proposes that human-AI collaboration modes have significantly different effects on eudaimonic well-being.

#### 3.2.1 Experiment 1 - preliminary experiment

Adopting the research design approach (Aaker et al., 2004), this study employs a combination of textual and visual materials to prime the situational experiment. Participants were instructed to carefully read the text and view the image stimuli to fully understand the experimental scenario before completing the corresponding measurement tasks.

The scenario was set as an important long-distance driving task. In the human-dominant mode, the AI system only provided basic navigation information, and all driving operations and responsibilities were fully undertaken by the participant. In the human-AI collaboration mode, the AI offered assistance and suggestions at critical moments, whereas participants retained final decision-making authority and were still responsible for most driving operations. In the AI-dominant mode, the AI took over nearly the entire driving process, leaving participants to passively accept the outcomes.

The stimulus materials for the three experimental groups are presented in the Supplementary Appendix.

At the beginning of the experiment, participants were randomly assigned to one of the three experimental conditions and required to complete the questionnaire after reading the corresponding materials. In terms of measurement instruments, established Likert scales were adopted and adapted to fit the experimental scenario, as presented in the Supplementary Appendix.

This study first conducted a pre-experiment to manipulate the three human-AI collaboration modes (human-dominant, human-AI collaboration, and AI-dominant) and examine the validity of the manipulation, ensuring that the three modes could be clearly distinguished within the experimental context. A total of 60 participants were recruited via the Credamo platform. After excluding invalid responses due to attention checks, excessively long or short completion time, and straight-lined answering, 51 valid samples remained. Reliability and validity tests were satisfactory (Cronbach’s α = 0.977, KMO = 0.908). A one-way ANOVA revealed significant differences in perceived AI involvement across the three experimental conditions, *F*(2, 48) = 171.192, *p* < 0.001. *Post-hoc* tests indicated significant pairwise differences among the human-dominant group (*M* = 1.659, SD = 0.643), the human-AI collaboration group (*M* = 2.459, SD = 1.153), and the AI-dominant group (*M* = 6.365, SD = 0.481), all *p* < 0.05.

#### 3.2.2 Experiment 1 - formal experiment

A total of 130 questionnaires were collected, and 125 valid samples were retained. Following the completion of formal data collection for Experiment 1, we adopted G*Power 3.1 to evaluate the statistical power of its significance tests. Experiment 1 employed a one-way between-subjects design with three groups. Holding α = 0.05 and statistical power at 0.80, the minimum detectable effect size supported by this sample size was *f* = 0.281. Accordingly, the sample size of Experiment 1 is sufficient to detect between-group differences in eudaimonic well-being across distinct human-AI collaboration modes.

Reliability and validity tests yielded satisfactory results (Cronbach’s α = 0.895; KMO = 0.871). A one-way ANOVA demonstrated that human-AI collaboration modes exerted a significant effect on eudaimonic well-being, with a statistically significant difference [*F*(2, 122) = 22.364, *p* < 0.001].

Games–Howell comparisons showed that eudaimonic well-being was significantly higher in the human-AI collaborative mode than in the human-dominant mode, mean difference = 0.543, *p* = 0.008, 95% CI [0.122, 0.963]. The collaborative condition also produced significantly higher eudaimonic well-being than the AI-dominant condition, mean difference = 1.617, *p* < 0.001, 95% CI [0.961, 2.274]. Moreover, eudaimonic well-being was significantly higher in the human-dominant condition than in the AI-dominant condition, mean difference = 1.075, *p* < 0.001, 95% CI [0.422, 1.727].

To further test the proposed nonlinear relationship, the three collaboration conditions were coded as −1 for the human-dominant condition, 0 for the human-AI collaborative condition, and 1 for the AI-dominant condition. The linear and quadratic terms were entered simultaneously into the regression model. The overall model was significant, *F*(2, 122) = 22.364, *p* < 0.001, R^2^ = 0.268, adjusted R^2^ = 0.256, indicating that the linear and quadratic terms jointly explained 26.8% of the variance in eudaimonic well-being.

The linear term was negative and significant, *b* = −0.537, SE = 0.125, *t* = −4.310, *p* < 0.001, 95% CI [−0.784, −0.291]. More importantly, the quadratic term was also negative and statistically significant, *b* = −1.080, SE = 0.215, *t* = −5.023, *p* < 0.001, 95% CI [−1.506, −0.654]. The estimated regression equation was:


$$\text{Y}\;=\;5.348-0.537\text{X}-1.080{\text{X}}^{2}$$

The significant negative quadratic coefficient indicates a concave relationship between human-AI collaboration mode and eudaimonic well-being. Consistent with the descriptive results, eudaimonic well-being increased from the human-dominant condition to the collaborative condition and then declined substantially in the AI-dominant condition. Thus, eudaimonic well-being reached its highest observed level under the human-AI collaborative mode, supporting H1. The quadratic relationship observed in Experiment 1 is illustrated in Figure 1.

**FIGURE 1:**
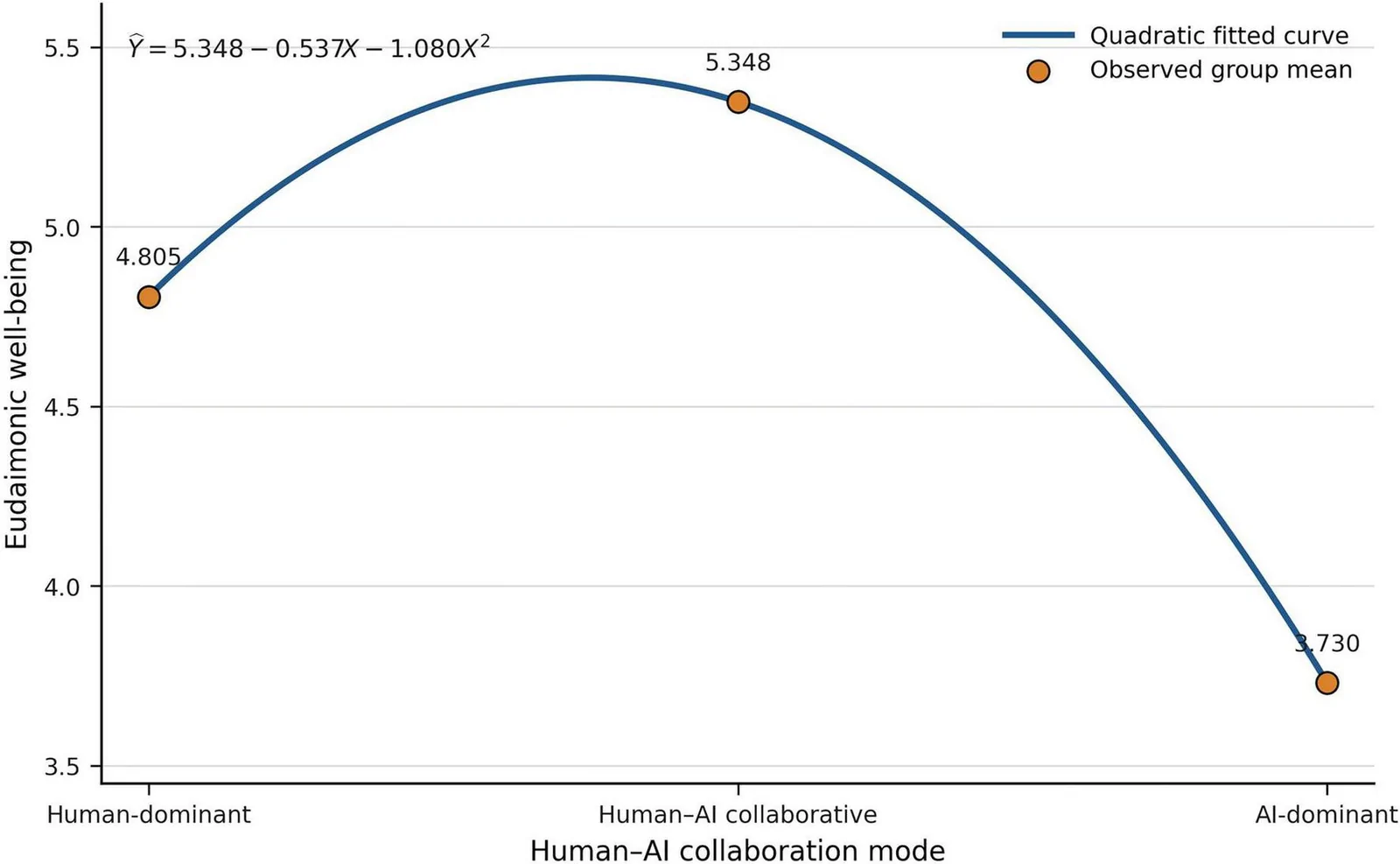
Effects of human-AI collaboration modes on eudaimonic well-being.

### 3.3 Experiment 2

Following the verification of the main effect of human-AI collaboration mode on eudaimonic well-being (Y) in Experiment 1, Experiment 2 re-examined H1 and further tested H2–H5.

#### 3.3.1 Experiment 2 - preliminary experiment

The pre-experiment adopted an online consumption decision-making task as the experimental scenario. Participants were asked to purchase a new laptop on an e-commerce platform with a reasonable but unfixed budget, aiming to meet the needs of study, work, and daily leisure entertainment. A variety of laptop brands, configurations and functional orientations were available on the market, with substantial differences in parameters and prices.

In the human-dominant mode, the AI only provided basic information and filtering tools, while participants maintained full control over decision-making and operational processes. In the human-AI collaboration mode, the AI offered several recommendations with detailed explanations. Participants could engage in in-depth collaboration, jointly adjust options, and make the final decision. In the AI-dominant mode, the system automatically generated and locked the recommended scheme, which participants were not allowed to modify, though humans still made the final confirmation decision.

The stimulus materials for the three experimental groups are presented in the Supplementary Appendix. Similar to Study 1, this study adopted well-established scales that were adapted to fit the experimental scenario.

This study first conducted a pre-experiment to manipulate the three human-AI collaboration modes, so as to test the effectiveness of the manipulation and ensure that the three modes could be clearly distinguished in the experimental scenario.

A total of 60 participants were recruited via the Credamo platform. Twelve invalid responses that failed the attention check were excluded. Reliability and validity tests yielded satisfactory results (Cronbach’s α = 0.954, KMO = 0.881).

A one-way ANOVA revealed significant differences in perceived AI involvement across experimental conditions [*F*(2, 45) = 43.146, *p* < 0.001]. *Post-hoc* tests further indicated significant pairwise differences among the human-dominant group (*M* = 1.863, SD = 0.768), the human-AI collaboration group (*M* = 2.825, SD = 1.001), and the AI-dominant group (*M* = 5.438, SD = 1.488), all at *p* < 0.05.

#### 3.3.2 Experiment 2 - formal experiment

The formal experiment used the same scenarios and manipulations as the pretest. A total of 269 valid participants completed the study: 83 in the human-dominant mode, 93 in the human-AI collaborative mode, and 93 in the AI-dominant mode. A sensitivity analysis conducted using G*Power 3.1 indicated that, for a three-group one-way ANOVA with α = 0.05 and statistical power of 0.80, the final sample could detect an effect of approximately *f* = 0.19. The study therefore had adequate power to identify small-to-medium between-group effects.

The internal consistency of the three focal constructs was assessed before hypothesis testing. Cronbach’s alpha was 0.874 for AI dependence, 0.708 for perceived autonomy, and 0.824 for eudaimonic well-being. These results indicated acceptable internal consistency for all three measures.

Descriptive statistics showed that participants in the human-AI collaborative condition reported the highest level of eudaimonic well-being (*n* = 93, *M* = 5.822, SD = 0.605), followed by those in the human-dominant condition (*n* = 83, *M* = 5.557, SD = 0.714). Participants in the AI-dominant condition reported the lowest level of eudaimonic well-being (*n* = 93, *M* = 5.056, SD = 1.144).

A conventional one-way ANOVA revealed a significant difference across the three conditions, *F*(2, 266) = 19.053, *p* < 0.001, η^2^ = 0.125. However, Levene’s test was significant, *F*(2, 266) = 14.431, *p* < 0.001, indicating that the homogeneity-of-variance assumption was violated. A Welch ANOVA and Games–Howell *post hoc* comparisons were therefore conducted. The Welch test remained significant, *F*(2, 168.28) = 16.728, *p* < 0.001.

Games–Howell comparisons showed that eudaimonic well-being was significantly higher in the human-AI collaborative condition than in the human-dominant condition, mean difference = 0.265, *p* = 0.025, 95% CI [0.028, 0.502]. The collaborative condition also produced significantly higher eudaimonic well-being than the AI-dominant condition, mean difference = 0.766, *p* < 0.001, 95% CI [0.448, 1.083]. Moreover, eudaimonic well-being was significantly higher in the human-dominant condition than in the AI-dominant condition, mean difference = 0.501, *p* = 0.002, 95% CI [0.164, 0.837].

To further examine the proposed nonlinear relationship, the three conditions were coded as −1 for the human-dominant condition, 0 for the human-AI collaborative condition, and 1 for the AI-dominant condition. The linear and quadratic terms were entered simultaneously into the regression model. The overall model was significant, *F*(2, 266) = 19.053, *p* < 0.001, R^2^ = 0.125, adjusted R^2^ = 0.119.

The linear coefficient was negative and significant, *b* = −0.250, SE = 0.065, *t* = −3.865, *p* < 0.001, 95% CI [−0.378, −0.123]. More importantly, the quadratic coefficient was also negative and significant, *b* = −0.515, SE = 0.110, *t* = −4.682, *p* < 0.001, 95% CI [−0.732, −0.299]. The estimated regression equation was:


$$Y=5.822-0.250\,X-0.515\,X^{2}$$

The significant negative quadratic coefficient indicated a concave relationship between human-AI collaboration mode and eudaimonic well-being. Consistent with the descriptive results, eudaimonic well-being increased from the human-dominant condition to the collaborative condition and subsequently decreased in the AI-dominant condition. Thus, eudaimonic well-being reached its highest observed level under the human-AI collaborative mode, supporting H1.

To test H2, quadratic regression analysis was conducted with AI dependence as the dependent variable. AI dependence was highest in the human-AI collaborative condition (*M* = 5.249, SD = 0.739), followed by the AI-dominant condition (*M* = 4.589, SD = 1.244) and the human-dominant condition (*M* = 4.014, SD = 1.400).

The quadratic regression model was significant, *F*(2, 266) = 25.312, *p* < 0.001, R^2^ = 0.160. The linear coefficient was positive and significant, *b* = 0.287, SE = 0.087, *t* = 3.302, *p* = 0.001, 95% CI [0.116, 0.459]. More importantly, the quadratic coefficient was negative and significant, *b* = −0.948, SE = 0.148, *t* = −6.409, *p* < 0.001, 95% CI [−1.239, −0.656]. The estimated equation was:


$$\mathrm{M1}=5.249+0.287\,X-0.948\,X^{2}$$

These results indicated that AI dependence reached its highest level under the human-AI collaborative mode and was lower under both the human-dominant and AI-dominant modes. H2 was therefore supported. The quadratic relationships for eudaimonic well-being and AI dependence in Experiment 2 are illustrated in Figure 2.

**FIGURE 2:**
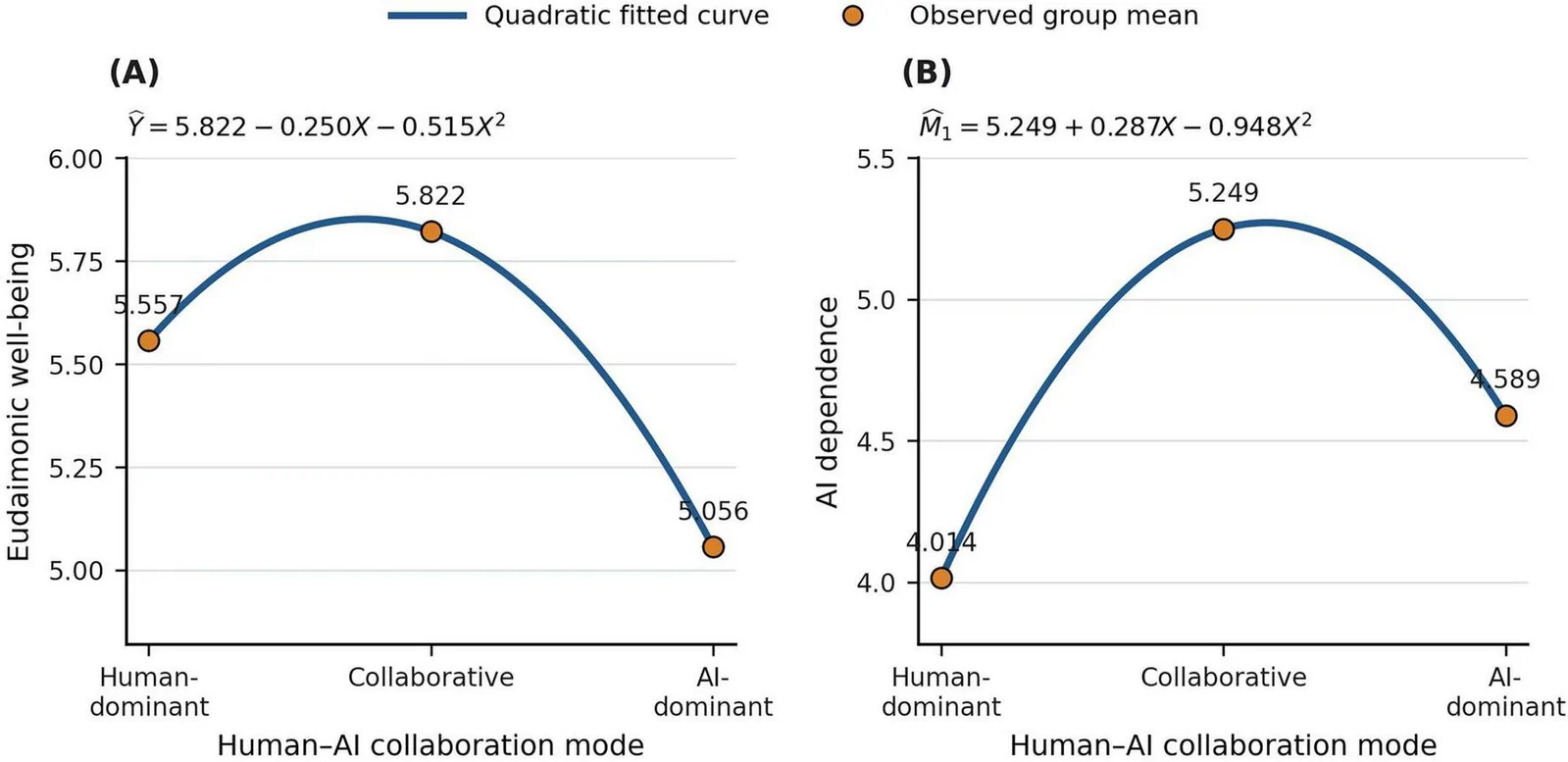
Quadratic effects of human-AI collaboration mode on eudaimonic well-being and AI dependence. **(A)** Quadratic relationship between human-AI collaboration mode and eudaimonic well-being. **(B)** Quadratic relationship between human-AI collaboration mode and AI dependence.

The regression model predicting perceived autonomy was significant, *F*(3, 265) = 18.229, *p* < 0.001, R^2^ = 0.171. AI dependence positively predicted perceived autonomy, *b* = 0.208, SE = 0.036, *t* = 5.757, *p* < 0.001, 95% CI [0.137, 0.279]. After controlling for AI dependence, perceived autonomy did not differ significantly between the human-dominant and collaborative conditions, *b* = −0.047, SE = 0.112, *p* = 0.673, whereas the AI-dominant condition produced significantly lower perceived autonomy than the collaborative condition, *b* = −0.317, SE = 0.102, *p* = 0.002. H3 was therefore supported.

The final regression model predicting eudaimonic well-being was significant, *F*(4, 264) = 107.198, *p* < 0.001, R^2^ = 0.619. Perceived autonomy positively predicted eudaimonic well-being, *b* = 0.784, SE = 0.051, *t* = 15.221, *p* < 0.001, 95% CI [0.682, 0.885]. AI dependence also positively predicted eudaimonic well-being, *b* = 0.155, SE = 0.032, *t* = 4.828, *p* < 0.001, 95% CI [0.092, 0.218]. H4 was therefore supported.

The serial mediating effects of AI dependence and perceived autonomy were examined using 5,000 bootstrap samples, with the human-AI collaborative condition serving as the reference category. For the comparison between the human-dominant and collaborative conditions, the serial indirect effect through AI dependence and perceived autonomy was significant, effect = −0.201, Boot SE = 0.058, 95% Bootstrap CI [−0.324, −0.103].

For the comparison between the AI-dominant and collaborative conditions, the serial indirect effect was also significant, effect = −0.107, Boot SE = 0.039, 95% Bootstrap CI [−0.196, −0.045]. Neither confidence interval contained zero. These findings demonstrated that AI dependence and perceived autonomy sequentially mediated the relationship between human-AI collaboration mode and eudaimonic well-being. H5 was therefore supported.

## 4 Research conclusions and discussion

### 4.1 Human-AI collaboration mode and users’ eudaimonic well-being

Based on the results of two formal experiments, this study finds a significant inverted U-shaped relationship between human-AI collaboration modes and eudaimonic well-being. This result supports Hypothesis 1 and aligns with the contingency perspective of Human-AI task allocation (Parasuraman et al., 2000).

Existing literature shows that an unbalanced ratio of Human-AI automation involvement significantly impairs users’ subjective experience in intelligent service consumption scenarios (Castelo et al., 2023; Longoni et al., 2019; Panagiotopoulos and Dimitrakopoulos, 2018). Under the human-dominant mode, insufficient functional support from algorithms increases users’ cognitive load and task burden. Under the AI-dominant mode, excessive algorithmic intervention restricts users’ decision-making autonomy and triggers negative psychological responses. A moderately balanced human-AI collaboration mode can both obtain algorithmic empowerment and retain users’ core decision-making control, thereby more effectively improving eudaimonic well-being.

By controlling irrelevant situational factors, this study further verifies that the above inverted U-shaped relationship is not caused by situational noise, but driven by systematic differences in Human-AI authority allocation within consumption task scenarios.

### 4.2 The chain mediating role of AI dependence and perceived autonomy

The results of this study indicate that AI dependence and perceived autonomy play a chain mediating role between human-AI collaboration modes and eudaimonic well-being. Mechanistically, graded human-AI collaboration modes first influence individuals’ AI dependence, then affect their perceived autonomy in the task scenario, and finally impact eudaimonic well-being.

Combined with the inverted U-shaped effect identified earlier, a moderately balanced human-AI collaboration mode can promote appropriate instrumental reliance on AI without excessively restricting individuals’ decision-making space. This helps maintain a high level of perceived autonomy and positively improves eudaimonic well-being. In contrast, insufficient AI support under the human-dominant mode and excessive restriction of perceived autonomy under the AI-dominant mode both interrupt this positive chain mediation pathway.

The findings reveal a sequential mechanism linking technology dependence, psychological autonomy, and long-term well-being in human-AI collaboration and address the lack of empirical research on mediation paths in the field of human-AI collaboration. The results provide empirical references for Human-AI authority allocation, collaboration rhythm regulation, and reducing Well-Being loss caused by technology overload or insufficient support in intelligent consumption scenarios.

### 4.3 Theoretical contributions

Taking autonomous driving and e-commerce decision-making as research scenarios, this study examines the inverted U-shaped relationship between human-AI collaboration modes (categorized as human-dominant, human-AI collaboration, and AI-dominant) and eudaimonic well-being.

Centering on the transfer of decisional control in human-AI interaction, this study engages in a threefold theoretical dialogue with prior literature. First, our findings respond to the challenge of realizing human-AI complementarity highlighted by Hemmer et al. (2025). The inverted U-shaped pattern shows that increasing AI involvement does not necessarily translate into greater eudaimonic well-being; rather, the well-being consequences of collaboration depend on how decisional authority is allocated between humans and AI. Second, we extend automation-bias research beyond cognitive and performance outcomes by showing that the allocation of decision authority can also shape users’ well-being through AI dependence and perceived autonomy. Third, we enrich the multi-level framework proposed by Puerta-Beldarrain et al. (2025) by incorporating distinct psychological mechanism accounts.

Our theoretical contributions are threefold. First, we identify and validate for the first time an inverted U-shaped relationship between human-AI collaboration modes and eudaimonic well-being, broadening the outcome variables of collaboration research from performance to well-being. Second, we develop and test a serial mediation model (collaboration mode–AI dependence–perceived autonomy–well-being), which integrates complementarity theory and automation bias research, positioning perceived autonomy as the pivotal hub that delineates the boundary between gain and loss. Third, we advance the contextualization of Self-determination theory by arguing that autonomy faces a differential threat in AI scenarios, establishing it as the most diagnostically valuable key mediator and thereby achieving theoretical sharpening of SDT in technology contexts.

### 4.4 Managerial implications

This study provides practical managerial implications for AI development, intelligent e-commerce platforms, algorithm system operation and intelligent service providers.

First, the design of AI development and system operation should adopt a moderately balanced allocation of responsibilities in human-AI collaboration. It is necessary to set an appropriate level of algorithm intervention. While giving full play to the effectiveness of AI tools, platforms should guarantee users’ decision-making dominance and maintain a balance between AI dependence and perceived autonomy.

Second, when optimizing recommendation algorithms and consumption decision-aided systems, enterprises can continuously monitor users’ level of AI dependence and perceived autonomy. A dynamically adjustable human-AI collaboration mechanism can be established to regulate algorithm recommendation frequency and intervention intensity. This approach avoids insufficient assistance or excessive algorithmic intervention. These implications can be translated into four concrete design principles. First, transparency requires systems to disclose when and why AI intervenes, the data and assumptions underlying consequential recommendations, and the uncertainty or limitations of the output. Second, controllability requires meaningful user controls, including the ability to modify goals, reject or revise recommendations, adjust intervention intensity, and readily return to human-led decision-making. Third, adaptive autonomy requires assistance to vary with task risk, user expertise, demonstrated reliance, and signs of cognitive overload, while avoiding covert personalization that silently transfers decision authority to the system. Fourth, a user-centered collaborative architecture should assign AI to information-intensive and reversible subtasks while reserving value-laden judgments and final authorization for users, supported by feedback channels, override logs, and periodic checks of perceived autonomy and Well-Being. Together, these principles operationalize human-centered AI as an auditable allocation of authority rather than a general call for more collaboration.

Finally, based on the psychological transmission mechanism identified in this study, enterprises can incorporate Well-Being experience optimization into the design process of intelligent services. Human-AI collaboration strategies can be adjusted according to differences among user groups. Such measures reduce negative experiences such as decision fatigue and declined sense of autonomy, and improve user satisfaction and long-term usage intention. These measures further promote the coordinated improvement of platform service quality, user Well-Being and user stickiness.

### 4.5 Limitations

Although this study follows standard experimental procedures and rigorous statistical paradigms, and verifies the core sequential mediation mechanism as well as the inverted U-shaped relationship in human-AI collaboration, several limitations still remain.

This research focuses on the empowering human-AI collaboration scenario. Undoubtedly, addictive AI dependence represents another important dimension of human-AI collaboration. Therefore, the external validity and generalizability of the research conclusions await further examination.

In addition, this study adopts cross-sectional questionnaires and retrospective self-reported data. It only captures users’ instantaneous psychological states in a single decision-making task from a static perspective, and cannot dynamically track the long-term changes of AI dependence, perceived autonomy and eudaimonic well-being. Thus, it is difficult to reveal the temporal dynamic causal relationships among these variables.

Furthermore, this study mainly concentrates on the core sequential mediation path. Moderating variables such as algorithm transparency, Human-AI emotional compatibility and algorithm trust are not incorporated. The marginal effects and interaction effects of multiple factors in complex human-AI collaboration scenarios remain unanalyzed.

### 4.6 Future directions

Future research can be advanced along four interrelated avenues.

First, test competing mediation mechanisms among the three SDT psychological needs. While the present study focuses on the autonomy pathway, competence and relatedness may also play dominant roles under different circumstances. Future work could specify parallel mediation models to systematically compare the relative weights of these three pathways, thereby clarifying the conditions under which the autonomy path is strengthened or attenuated. For instance, when AI demonstrates superhuman capabilities in professional domains (e.g., medical diagnosis, legal reasoning), the gain effect through competence may outweigh the loss through autonomy; likewise, in highly anthropomorphic interaction contexts, the relatedness pathway may become salient.

Second, expand the breadth of research contexts and examine cross-cultural heterogeneity. Extending the scenarios to creative generation, health-related decision-making, and AI companionship would enhance the external validity of our findings. Moreover, cross-cultural comparisons could uncover cultural variations in sensitivity to control delegation, providing calibration guidelines for the global design of human-AI collaboration.

Third, adopt longitudinal designs to capture the dynamic evolution of AI dependence. By combining diary methods or experience sampling with objective platform logs, researchers can trace the phase transition from empowering dependence to addictive dependence, identify critical tipping points at which perceived autonomy reverses, and thus reveal the dynamic regularities underlying the well-being effects of human-AI collaboration.

Fourth, integrate multi-level moderators to map boundary conditions. Moderation effects can be examined from technological (algorithmic transparency), individual (AI literacy), and task (task complexity) perspectives, assessing how these factors influence the inverted U-shaped relationship and the strength of the mediating pathways. In addition, the double-edged nature of AI anthropomorphism merits further investigation. Whether the fulfillment of relatedness can offset autonomy erosion depends on the optimal balance between anthropomorphic and instrumental attributes, a critical issue for responsible AI design.

## Data Availability

The original contributions presented in this study are included in this article/Supplementary material, further inquiries can be directed to the corresponding authors.

## References

[B1] Aaker J. L. Fournier S. Brasel S. A. (2004). When good brands do bad. *J. Consum. Res.* 31, 1–16. 10.1086/383419 32133228 PMC7041482

[B2] Afroogh S. Akbari A. Malone E. Kargar M. Alambeigi H. (2024). Trust in AI: Progress, challenges, and future directions. *Human. Soc. Sci. Commun.* 11:1568. 10.1057/s41599-024-04044-8

[B3] Ahmad R. Nawaz M. R. Ishaq M. I. Khan M. M. Ashraf H. A. (2023). Social exchange theory: Systematic review and future directions. *Front. Psychol.* 13:1015921. 10.3389/fpsyg.2022.1015921 36710813 PMC9878386

[B4] Azeez M. A. Siddiqui Z. H. Sohail S. S. (2023). ChatGPT charting the course: Artificial intelligence in healthcare—Insights and future prospects. *Cancer Res. Statist. Treatment* 6, 630–631. 10.4103/crst.crst_327_23 36879322

[B5] Bahoo S. Cucculelli M. Qamar D. (2023). Artificial intelligence and corporate innovation: A review and research agenda. *Technol. Forecast. Soc. Change* 188:122264. 10.1016/j.techfore.2022.122264

[B6] Bauer E. Greiff S. Graesser A. C. Scheiter K. Sailer M. (2025). Looking beyond the hype: Understanding the effects of AI on learning. *Educ. Psychol. Rev.* 37:45. 10.1007/s10648-025-10020-8

[B7] Bayor L. Weinert C. Maier C. Weitzel T. (2025). Social-Oriented communication with AI companions: Benefits, costs, and contextual patterns. *Bus. Inform. Syst. Eng.* 67, 637–655. 10.1007/s12599-025-00955-1

[B8] Bhardwaj S. Jain V. Mahapatra D. Sindhwani R. (2025). Exploring the dark side of AI and its influence on consumer emotion. *J. Consumer Behav.* 24, 529–544. 10.1002/cb.2431

[B9] Castelo N. Boegershausen J. Hildebrand C. Henkel A. P. (2023). Understanding and improving consumer reactions to service bots. *J. Consumer Res.* 50, 848–863. 10.1093/jcr/ucad023

[B10] Chen O. Paas F. Sweller J. (2023). A cognitive load theory approach to defining and measuring task complexity through element interactivity. *Educ. Psychol. Rev.* 35:63. 10.1007/s10648-023-09782-w

[B11] Chrisinger B. W. Gustafson J. A. King A. C. Winter S. J. (2019). Understanding where we are well: Neighborhood-level social and environmental correlates of well-being in the stanford well for life study. *Int. J. Environ. Res. Public Health* 16:1786. 10.3390/ijerph16101786 31137589 PMC6571676

[B12] Darling-Hammond L. Flook L. Cook-Harvey C. Barron B. Osher D. (2020). Implications for educational practice of the science of learning and development. *Appl. Dev. Sci.* 24, 97–140. 10.1080/10888691.2018.1537791

[B13] Davlembayeva D. Papagiannidis S. Alamanos E. (2020). Sharing economy: Studying the social and psychological factors and the outcomes of social exchange. *Technol. Forecast. Soc. Change* 158:120143. 10.1016/j.techfore.2020.120143

[B14] Deci E. L. Olafsen A. H. Ryan R. M. (2017). Self-Determination theory in work organizations: The state of a science. *Ann. Rev. Organ. Psychol. Organ. Behav.* 4, 19–43. 10.1146/annurev-orgpsych-032516-113108

[B15] Denecke K. Gabarron E. Grainger R. Konstantinidis S. T. Lau A. Rivera-Romero O. et al. (2019). Artificial intelligence for participatory health: Applications, impact, and future implications. *Yearb. Med. Inform.* 28, 165–173. 10.1055/s-0039-1677902 31022749 PMC6697496

[B16] Dietvorst B. J. Simmons J. P. Massey C. (2015). Algorithm aversion: People erroneously avoid algorithms after seeing them err. *J. Exp. Psychol. Gen.* 144, 114–126. 10.1037/xge0000033 25401381

[B17] Ding S. Pan X. Hu L. Liu L. A. (2025). new model for calculating human trust behavior during human-AI collaboration in multiple decision-making tasks: A Bayesian approach. *Comp. Indust. Eng.* 200:110872. 10.1016/j.cie.2025.110872

[B18] Edwards J. Nguyen A. Lämsä J. Sobocinski M. Whitehead R. Dang B. et al. (2025). Human- AI collaboration: Designing artificial agents to facilitate socially shared regulation among learners. *Br. J. Educ. Technol.* 56, 712–733. 10.1111/bjet.13534

[B19] Ekin M. Krejtz K. Krejtz I. (2025). Cognitive load and oculometrics in the educational scenarios: A systematic review and meta-analysis. *Proc. ACM Human-Comp. Interact.* 9, 1–19. 10.1145/3725832

[B20] Filippelli S. Popescu I. A. Verteramo S. Tani M. Corvello V. (2026). Generative AI and employee well-being: Exploring the emotional, social, and cognitive impacts of adoption. *J. Innov. Knowledge* 11:100844. 10.1016/j.jik.2025.100844

[B21] Frank D. Folwarczny M. Otterbring T. (2026). Consumer acceptance of high-autonomy AI assistants is driven by perceived benefits in online shopping settings characterized by scarcity. *Psychol. Market.* 43, 538–555. 10.1002/mar.70074

[B22] Frenkenberg A. Hochman G. (2025). It’s scary to use it, it’s scary to refuse it: The psychological dimensions of AI adoption—anxiety, motives, and dependency. *Systems* 13:82. 10.3390/systems13020082

[B23] Gagné M. Deci E. L. (2005). Self-determination theory and work motivation. *J. Organ. Behav.* 26, 331–362. 10.1002/job.322

[B24] Gao J. Wang H. Li W. Li G. He S. (2025). Research on the effect of the empathy ability of AI service robots on consumer well-being: The chain mediating model. *Intern. J. Human–Comp. Interact.* 41, 6716–6728. 10.1080/10447318.2024.2383483

[B25] Hancock P. A. Kessler T. T. Kaplan A. D. Stowers K. Brill J. C. Billings D. R. et al. (2023). How and why humans trust: A meta-analysis and elaborated model. *Front. Psychol.* 14:1081086. 10.3389/fpsyg.2023.1081086 37051611 PMC10083508

[B26] Hemmer P. Schemmer M. Kühl N. Vössing M. Satzger G. (2025). Complementarity in Human-AI collaboration: Concept, sources, and evidence. *Eur. J. Inform. Syst.* 34, 979–1002. 10.1080/0960085X.2025.2475962

[B27] Hou H. Lin S. Ni S. Wang P. (2024). When AI learns to empathize: Topics, scenarios, and optimization of empathy computing from a psychological perspective. *Adv. Psychol. Sci.* 32:845. 10.3724/sp.j.1042.2024.00845

[B28] Howard Z. L. Evans N. J. Innes R. J. Brown S. D. Eidels A. (2020). How is multi-tasking different from increased difficulty? *Psychon. Bull. Rev.* 27, 937–951. 10.3758/s13423-020-01741-8 32440999

[B29] Hsu C. Lin J. C. (2023). Understanding the user satisfaction and loyalty of customer service chatbots. *J. Retail. Consumer Serv.* 71:103211. 10.1016/j.jretconser.2022.103211

[B30] Huang Y. Chuang N. Kwok L. (2023). To speak up or remain silent: The double-edged effects of trust and felt trust. *Intern. J. Contemp. Hospital. Manag.* 35, 3285–3304. 10.1108/ijchm-05-2022-0676

[B31] Hunt A. Merola G. P. Carpenter T. Jaeggi A. V. (2024). Evolutionary perspectives on substance and behavioural addictions: Distinct and shared pathways to understanding, prediction and prevention. *Neurosci. Biobehav. Rev.* 159:105603. 10.1016/j.neubiorev.2024.105603 38402919

[B32] Iqbal A. Mansell W. (2021). A thematic analysis of multiple pathways between nature engagement activities and well-being. *Front. Psychol.* 12:580992. 10.3389/fpsyg.2021.580992 33841228 PMC8032872

[B33] Jeno L. M. Vandvik V. Eliassen S. Grytnes J. (2019). Testing the novelty effect of an m-learning tool on internalization and achievement: A self-determination theory approach. *Comp. Educ.* 128, 398–413. 10.1016/j.compedu.2018.10.008

[B34] Jia R. (2025). Generative AI in performance design: Type construction and current application status. *Commun. Human. Res.* 67, 24–28. 10.54254/2753-7064/2025.bo23977

[B35] Jiang Z. (2010). Exploring the Roles of Trust, Reliance and Commitment in Business Relationships. Manchester.

[B36] Jiao J. Cao X. (2024). Research on designers’ behavioral intention toward artificial intelligence-aided design: Integrating the theory of planned behavior and the technology acceptance model. *Front. Psychol.* 15:1450717. 10.3389/fpsyg.2024.1450717 39351117 PMC11439648

[B37] Kamar E. (2016). “Directions in Hybrid Intelligence: Complementing AI Systems with Human Intelligence,” in *Proceedings of the International Joint Conference on Artificial Intelligence*, (New York, NY: ACM).

[B38] Klingbeil A. Grützner C. Schreck P. (2024). Trust and reliance on AI — An experimental study on the extent and costs of overreliance on AI. *Comp. Hum. Behav.* 160:108352. 10.1016/j.chb.2024.108352

[B39] Laitinen A. Sahlgren O. A. I. (2021). Systems and respect for human autonomy. *Front. Artif. Intell.* 4:705164. 10.3389/frai.2021.705164 34765969 PMC8576577

[B40] Lan Y. Liu S. Chen H. (2026). Exploring the formation of learning burnout among college students in AI context: A serial mediation mechanism of AI dependence and addiction based on I-PACE model. *Front. Comp. Sci.* 8:1756441. 10.3389/fcomp.2026.1756441

[B41] Lee J. D. See K. A. (2004). Trust in automation: Designing for appropriate reliance. *Hum. Factors* 46, 50–80. 10.1518/hfes.46.1.50_30392 15151155

[B42] Lee S. A. Shin H. H. Lee M. Jeong M. (2025). Feelings matter in the digital age: The role of corporate digital responsibility on employee well-being and advocacy behavior. *Intern. J. Contemp. Hospital. Manag.* 37, 2327–2348. 10.1108/ijchm-04-2024-0586

[B43] Leng J. Zheng K. Li R. Chen C. Wang B. Liu Q. et al. (2026). AIGC-empowered smart manufacturing: Prospects and challenges. *Robot. Computer-Integr. Manufact.* 97:103076. 10.1016/j.rcim.2025.103076

[B44] Leonardelli G. J. Pickett C. L. Brewer M. B. (2010). “Optimal Distinctiveness Theory,” in *Advances in Experimental Social Psychology*, eds Zanna M. P. Olson J. M. (Amsterdam: Elsevier).

[B45] Li S. (2026). Partner or burden? The dual pathways linking perceived attributes of intelligent cockpits to human-machine collaboration willingness via cognitive load. *BMC Psychol.* 14:313. 10.1186/s40359-026-04074-8 41639926 PMC12964694

[B46] Li Y. Hou R. Tan R. (2024). How customers respond to chatbot anthropomorphism: The mediating roles of perceived humanness and perceived persuasiveness. *Eur. J. Market.* 58, 2757–2790. 10.1108/ejm-11-2022-0827

[B47] Longoni C. Bonezzi A. Morewedge C. K. (2019). Resistance to medical artificial intelligence. *J. Consumer Res.* 46, 629–650. 10.1093/jcr/ucz013

[B48] Lu J. Yan Y. Huang K. Yin M. Zhang F. (2024). Do we learn from each other: Understanding the Human-AI Co-Learning process embedded in Human-AI collaboration. *Group Decis. Negotiat.* 34, 235–271. 10.1007/s10726-024- 09912-x

[B49] Lyell D. Magrabi F. Coiera E. (2018). The effect of cognitive load and task complexity on automation bias in electronic prescribing. *Hum. Fact.* 60, 1008–1021. 10.1177/0018720818781224 29939764

[B50] Muhammad K. Ullah A. Lloret J. Ser J. D. de Albuquerque V. H. C. (2021). Deep learning for safe autonomous driving: Current challenges and future directions. *IEEE Trans. Intell. Trans. Syst.* 22, 4316–4336. 10.1109/tits.2020.3032227

[B51] Nadarzynski T. Miles O. Cowie A. Ridge D. (2019). Acceptability of artificial intelligence (AI)-led chatbot services in healthcare: A mixed-methods study. *Digit Health* 5:2055207619871808. 10.1177/2055207619871808 31467682 PMC6704417

[B52] Nambisan S. Lyytinen K. Majchrzak A. Song M. (2017). Digital innovation management: Reinventing innovation management research in a digital world. *MIS Quarterly* 41, 223–238. 10.25300/misq/2017/41:1.03

[B53] Nayyar A. Maqsood A. (2026). From human bonds to artificial support: Perceived AI empathy, dependence on AI chatbots, and social disconnection among emerging adults. *Qlantic J. Soc. Sci. Human.* 7:70. 10.55737/qjssh.vii-i.26463

[B54] Nazareno L. Schiff D. S. (2021). The impact of automation and artificial intelligence on worker well-being. *Technol. Soc.* 67:101679. 10.1016/j.techsoc.2021.101679

[B55] Niu W. Zhang W. Zhang C. Chen X. (2024). The role of artificial intelligence autonomy in higher education: A uses and gratification Perspective. *Sustainability* 16:1276. 10.3390/su16031276

[B56] Novrouzai I. (2025). *AI Vs HR-Led Well-Being Interventions and Employee Well-Being: The Mediating Role of Perceived Organizational Support.* Lisbon.

[B57] Oh J. Nah S. Yang Z. D. (2025). How autonomy of artificial intelligence technology and user agency influence AI perceptions and attitudes: Applying the theory of psychological reactance. *J. Broadcasting Electron. Med.* 69, 161–182. 10.1080/08838151.2025.2485319

[B58] Oh S. Kim J. Hongkiangko M. Kang C. Oh S. Jeong S. et al. (2024). Factors influencing Human-AI collaboration: A scoping review. *J. Korea Multimed. Soc.* 27, 945–958. 10.9717/kmms.2024.27.8.945

[B59] Pan M. Lai C. Guo K. (2025). Effects of GenAI-empowered interactive support on university EFL students’ self-regulated strategy use and engagement in reading. *Internet Higher Educ.* 65:100991. 10.1016/j.iheduc.2024.100991

[B60] Panagiotopoulos I. Dimitrakopoulos G. (2018). An empirical investigation on consumers’ intentions towards autonomous driving. *Trans. Res. Part C: Emerg. Technol.* 95, 773–784. 10.1016/j.trc.2018.08.013

[B61] Parasuraman R. Riley V. (1997). Humans and automation: Use, misuse, disuse, abuse. *Hum. Fact.* 39, 230–253. 10.1518/001872097778543886

[B62] Parasuraman R. Sheridan T. B. Wickens C. D. (2000). A model for types and levels of human interaction with automation. *IEEE Trans. Syst. Man Cybernetics - Part A: Syst. Hum.* 30, 286–297. 10.1109/3468.844354 11760769

[B63] Peng X. Sun K. Shan X. Zhang J. (2026). Empowerment or dependency? A systematic review of the impacts of intelligent assessment and generative AI on learners’ self-beliefs and cognitive agency in music education. *Front. Psychol.* 17:1776445. 10.3389/fpsyg.2026.1776445 41783302 PMC12953392

[B64] Pham H. C. Duong C. D. Nguyen G. K. H. (2024). What drives tourists’ continuance intention to use ChatGPT for travel services? A stimulus-organism-response perspective. *J. Retail. Consumer Serv.* 78:103758. 10.1016/j.jretconser.2024.103758

[B65] Pham T. (2025). Ethical and legal considerations in healthcare AI: Innovation and policy for safe and fair use. *R. Soc. Open Sci.* 12:241873. 10.1098/rsos.241873 40370601 PMC12076083

[B66] Puerta-Beldarrain M. Gómez-Carmona O. Sánchez-Corcuera R. Casado-Mansilla D. López-de-Ipiña D. Chen L. A. (2025). Multifaceted vision of the Human-AI collaboration: A comprehensive review. *IEEE Access* 13, 29375–29405. 10.1109/access.2025.3536095

[B67] Pyke W. Lunau J. Javadi A. H. (2025). Does difficulty moderate learning? A comparative analysis of the desirable difficulties framework and cognitive load theory. *Q. J. Exp. Psychol.* 78, 2181–2195. 10.1177/17470218241308143 39641213 PMC12432286

[B68] Raman R. Gunasekaran A. Suresh M. (2026). Enabling flexible, resilient, and sustainable supply chains through metaverse technologies. *Global J. Flexible Syst. Manag.* 27, 345–370. 10.1007/s40171-026-00490-2

[B69] Romeo G. Conti D. (2026). Exploring automation bias in Human–AI collaboration: A review and implications for explainable AI. *AI Soc.* 41, 259–278. 10.1007/s00146-025-02422-7

[B70] Ryan M. (2020). In AI we trust: Ethics, artificial intelligence, and reliability. *Sci. Eng. Ethics* 26, 2749–2767. 10.1007/s11948-020-00228-y 32524425 PMC7550313

[B71] Ryan R. M. Deci E. L. (2000). Self-determination theory and the facilitation of intrinsic motivation, social development, and well-being. *Am. Psychol.* 55, 68–78. 10.1037//0003-066x.55.1.68 11392867

[B72] Ryan R. M. Huta V. Deci E. L. (2008). Living well: A self-determination theory perspective on eudaimonia. *J. Happ. Stud.* 9, 139–170. 10.1007/s10902-006-9023-4

[B73] Saleh M. I. (2025). Generative artificial intelligence in hospitality and tourism: Future capabilities, AI prompts and real-world applications. *J. Hospital. Market. Manag.* 34, 467–498. 10.1080/19368623.2025.2458603

[B74] Sharma A. (2024). *Human-AI Collaboration to Support Mental Health and Well-Being.* New York, NY: Association for Computing Machinery.

[B75] Sharma A. Lin I. W. Miner A. S. Atkins D. C. Althoff T. (2023). Human–AI collaboration enables more empathic conversations in text-based peer-to-peer mental health support. *Nat. Mach. Intell.* 5, 46–57. 10.1038/s42256-022-00593-2

[B76] Sheldon K. M. Ryan R. M. Deci E. L. Kasser T. (2004). The independent effects of goal contents and motives on well-being: It’s both what you pursue and why you pursue it. *Pers. Soc. Psychol. Bull.* 30, 475–486. 10.1177/0146167203261883 15070476

[B77] Shimu S. J. Patil S. M. Dadzie E. Tesfaye T. Alag P. Więckiewicz G. (2024). Exploring health informatics in the battle against drug addiction: Digital solutions for the rising concern. *J. Pers. Med.* 14:556. 10.3390/jpm14060556 38929777 PMC11204661

[B78] Shin D. (2021). The effects of explainability and causability on perception, trust, and acceptance: Implications for explainable AI. *Intern. J. Human-Comp. Stud.* 146:102551. 10.1016/j.ijhcs.2020.102551

[B79] Steyvers M. Kumar A. (2024). Three challenges for AI-Assisted decision-making. *Perspect. Psychol. Sci.* 19, 722–734. 10.1177/17456916231181102 37439761 PMC11373149

[B80] Sweller J. (1988). Cognitive load during problem solving: Effects on learning. *Cogn. Sci.* 12, 257–285. 10.1207/s15516709cog1202_4 42769788

[B81] Topol E. J. (2019). High-performance medicine: The convergence of human and artificial intelligence. *Nat. Med.* 25, 44–56. 10.1038/s41591-018-0300-7 30617339

[B82] Vaccaro M. Almaatouq A. Malone T. (2024). When combinations of humans and AI are useful: A systematic review and meta-analysis. *Nat. Hum. Behav.* 8, 2293–2303. 10.1038/s41562-024-02024-1 39468277 PMC11659167

[B83] Vats V. Nizam M. B. Liu M. Wang Z. Ho R. Prasad M. S. et al. (2024). A survey on Human-AI collaboration with large foundation models. *arXiv [Preprint]* 10.48550/arXiv.2403.04931

[B84] Wang Z. Yuan R. Li B. (2025). Are recommendation systems annoying? An empirical study of assessing the impacts of AI characteristics on technology well-being. *J. Consumer Behav.* 24, 178–200. 10.1002/cb.2408

[B85] Waterman A. S. (1993). Two conceptions of happiness: Contrasts of personal expressiveness (eudaimonia) and hedonic enjoyment. *J. Personal. Soc. Psychol.* 64, 678–691. 10.1037/0022-3514.64.4.678

[B86] Weng Z. Huang Y. Weng S. (2026). From code to care: How artificial empathy enhances customer experience in human-robot interaction. *J. Bus. Res.* 206:115969. 10.1016/j.jbusres.2026.115969

[B87] Williams G. C. Rodin G. C. Ryan R. M. Grolnick W. S. Deci E. L. (1998). Autonomous regulation and long-term medication adherence in adult outpatients. *Health Psychol.* 17, 269–276. 10.1037/0278-6133.17.3.269 9619477

[B88] Wu T. Zhang R. Zhang Z. (2025). Navigating the human-artificial intelligence collaboration landscape: Impact on quality of work life and work engagement. *J. Hospital. Tour. Manag.* 62, 276–283. 10.1016/j.jhtm.2025.02.005

[B89] Yang H. Zhou Y. Wu J. Liu H. Yang L. Lv C. (2025). Human-Guided continual learning for personalized decision-making of autonomous driving. *IEEE Trans. Intell. Trans. Syst.* 26, 5435–5447. 10.1109/tits.2024.3524609

[B90] Yousef A. M. F. Alshamy A. Tlili A. Metwally A. H. S. (2025). Demystifying the New dilemma of brain rot in the digital era: A review. *Brain Sci.* 15:283. 10.3390/brainsci15030283 40149804 PMC11939997

[B91] Zhai C. Wibowo S. Li L. D. (2024). The effects of over-reliance on AI dialogue systems on students’ cognitive abilities: A systematic review. *Smart Learn. Environ.* 11:28. 10.1186/s40561-024-00316-7

[B92] Zhang J. Tao D. (2021). Empowering things with intelligence: A survey of the progress, challenges, and opportunities in artificial intelligence of things. *IEEE Internet Things J.* 8, 7789–7817. 10.1109/jiot.2020.3039359

[B93] Zhang S. Mahemuti M. Tang J. Han X. Li X. (2025). Avatars at the forefront: How collaboration types influence purchase intention. *Service Indust. J.* 45, 1313–1347. 10.1080/02642069.2025.2458213

[B94] Zhao Q. van Prooijen J. W. Jiang X. Spadaro G. (2025). Suspicious of AI? Perceived autonomy and interdependence predict AI-related conspiracy beliefs. *Br. J. Soc. Psychol.* 64:e12883. 10.1111/bjso.12883 40168138 PMC11960797

[B95] Zhou S. Yi N. Rasiah R. Zhao H. Mo Z. (2024). An empirical study on the dark side of service employees’ AI awareness: Behavioral responses, emotional mechanisms, and mitigating factors. *J. Retail. Consumer Serv.* 79:103869. 10.1016/j.jretconser.2024.103869

