# Supplementary material for: Too much, too little, or just right? The inverted-U relationship between human-AI collaboration modes and eudaimonic well-being
Source: Front Psychol. 2026 Sep 28;17:1747252. doi: 10.3389/fpsyg.2026.1747252 (PMC13655146; doi:10.3389/fpsyg.2026.1747252)
Supplement: Supplementary file 1 [file Supplementary_file_1.zip › Section X and Y.docx]

Section X

QA1 The AI system **controls** the decision-making process in this task.

QA2 Overall, the AI system **plays** a dominant role in completing the task.

QA3 Decisions in the task **are** mainly **made** by the AI system rather than by me.

QA4 During the decision process, I **feel** that the primary control **rests** with me (reverse coded).

QA5 The AI system’s involvement **determines** the final outcome of the task.

Section Y

Y1 I feel that this journey gives me a clear sense of direction and meaning in life.

Y2 I believe that I experience personal growth during this trip.

Y3 My behavior in this trip expresses my true self.

Y4 I engage in activities during this trip that are consistent with my beliefs.

Y5 Through this AI-assisted driving journey, I feel I am moving toward the realization of my full potential.
