# Supplementary material for: Too much, too little, or just right? The inverted-U relationship between human-AI collaboration modes and eudaimonic well-being
Source: Front Psychol. 2026 Sep 28;17:1747252. doi: 10.3389/fpsyg.2026.1747252 (PMC13655146; doi:10.3389/fpsyg.2026.1747252)
Supplement: Supplementary file 1 [file Supplementary_file_1.zip › Experiment 2 - Chinese.docx]

# 实验二

欢迎您作答本问卷。

请设想你正在为自己购买一台新笔记本电脑，预算适中，主要用于学习、工作与部分娱乐。你非常重视这次选购，希望在性能、品牌、价格、售后等方面做出权衡决策。为了提升效率，你选择在某智能电商平台使用AI推荐系统辅助完成选购任务。

3-版块开始

[图文说明]

请设想你正在为自己购买一台新笔记本电脑，预算适中，主要用于学习、工作与部分娱乐。你非常重视这次选购，希望在性能、品牌、价格、售后等方面做出权衡决策。为了提升效率，你选择在某智能电商平台使用AI推荐系统辅助完成选购任务。

AI系统要求你输入预算范围与主要用途后，将直接为你提供1款“综合评分最高”的机型推荐，并解释其依据（如数据排名、用户标签模型）。你无法修改推荐参数，也不能浏览其他商品页面。

整个过程中，**你无法干预决策过程或查看其他选项**，只可“确认购买”或“放弃本轮推荐”，AI以黑箱逻辑自动完成整个选购路径。

3-版块结束

2-版块开始

[图文说明]

请设想你正在为自己购买一台新笔记本电脑，预算适中，主要用于学习、工作与部分娱乐。你非常重视这次选购，希望在性能、品牌、价格、售后等方面做出权衡决策。为了提升效率，你选择在某智能电商平台使用AI推荐系统辅助完成选购任务。

平台上的AI系统会根据你的基础偏好与用途自动生成5款候选机型（含配置对比与理由说明）。你可修改筛选条件，或通过“问答式交互”告知系统你的偏好（如更轻薄、续航更强、价格范围等）。

AI系统会根据你的反馈**动态优化推荐结果**，并提供理由说明。你与AI像一个智能顾问与搭档共同完成任务，推荐结果可以被修改，你始终保持最终决策权。

2-版块结束

1-版块开始

[图文说明]

请设想你正在为自己购买一台新笔记本电脑，预算适中，主要用于学习、工作与部分娱乐。你非常重视这次选购，希望在性能、品牌、价格、售后等方面做出权衡决策。为了提升效率，你选择在某智能电商平台使用AI推荐系统辅助完成选购任务。

平台仅提供一个完整的笔记本商品数据库及筛选器（如价格、品牌、用途）。所有选项由你亲自设定，你需要手动比较多个机型的配置、性能、口碑与价格。平台不会主动给出推荐或建议，所有选择都由你自主完成。

整个过程中，**AI不会介入推荐或互动**，你需投入较多时间精力，依靠自己的经验做出决策。

1-版块结束
