# Supplementary material for: Too much, too little, or just right? The inverted-U relationship between human-AI collaboration modes and eudaimonic well-being
Source: Front Psychol. 2026 Sep 28;17:1747252. doi: 10.3389/fpsyg.2026.1747252 (PMC13655146; doi:10.3389/fpsyg.2026.1747252)
Supplement: Supplementary file 1 [file Supplementary_file_1.zip › Experiment 2 - English.docx]

Experiment 2

Welcome to complete this questionnaire.

Please imagine that you are purchasing a new laptop for yourself, with a moderate budget, mainly for study, work, and some entertainment. You place great importance on this purchase and wish to make a balanced decision considering performance, brand, price, and after-sales service. To improve efficiency, you have chosen to use an AI recommendation system on a smart e-commerce platform to assist you with the selection task.

Section 1 begins

Please envision that you are buying a new laptop for yourself with a moderate budget, mainly for study, work, and some entertainment. You take this purchase very seriously and want to make a balanced decision considering performance, brand, price, and after-sales service. To improve efficiency, you have chosen to use an AI recommendation system on a smart e-commerce platform to assist you with the selection task.

The platform only provides a complete laptop product database and a filter (such as price, brand, purpose). All options are set by you personally, and you need to manually compare the configurations, performance, reputation, and prices of multiple models. The platform will not actively give recommendations or suggestions; all choices are made independently by you.

Throughout the entire process, AI will not be involved in making recommendations or interacting; you will need to invest more time and effort and rely on your own experience to make decisions.

Section 1 ends

Section 2 begins

Please imagine that you are purchasing a new laptop for yourself, with a moderate budget, primarily for study, work, and some entertainment. You place great importance on this selection and hope to make a balanced decision considering performance, brand, price, and after-sales service. To enhance efficiency, you have chosen to use an AI recommendation system on a smart e-commerce platform to assist you with the purchasing task.

The AI system on the platform will automatically generate 5 candidate models (including configuration comparisons and reasons) based on your basic preferences and usage. You can modify the filtering criteria or inform the system of your preferences through "question-and-answer interactions" (such as being more lightweight, having longer battery life, price range, etc.).

The AI system will dynamically optimize the recommendation results based on your feedback and provide reasons for the changes. You and the AI work together like an intelligent consultant and partner to complete the task, and the recommendations can be modified; you always retain the final decision-making power.

Section 2 ends

Section 3 begins

Please envision that you are buying a new laptop for yourself with a moderate budget, primarily for study, work, and some entertainment. You take this purchase very seriously and wish to make a balanced decision considering performance, brand, price, and after-sales service. To increase efficiency, you have chosen to use an AI recommendation system on a smart e-commerce platform to assist you in completing the purchase task.

The AI system requires you to input your budget range and main purpose, and then it will directly provide you with a recommendation for one model with the "highest overall score," explaining its basis (such as data rankings, user tag models). You cannot modify the recommendation parameters, nor can you browse other product pages.

Throughout the entire process, you cannot intervene in the decision-making process or view other options; you can only "confirm the purchase" or "abandon this round of recommendations." The AI automatically completes the entire purchase path with a black-box logic.

Section 3 ends
