# Supplementary material for: Too much, too little, or just right? The inverted-U relationship between human-AI collaboration modes and eudaimonic well-being
Source: Front Psychol. 2026 Sep 28;17:1747252. doi: 10.3389/fpsyg.2026.1747252 (PMC13655146; doi:10.3389/fpsyg.2026.1747252)
Supplement: Supplementary file 1 [file Supplementary_file_1.zip › Experiment 1 - Chinese.docx]

# 实验一-预实验

欢迎您作答本问卷。

请设想你正在进行一段重要的长途自驾旅程，从成都前往西安（全程约650公里，时长约8小时）。

1-版块开始

Q1 [图文说明]

您正独自驾驶一辆搭载传统导航系统的车辆，系统只能提供基本路线导航信息（如红绿灯位置、转弯提示），不具备任何自动驾驶功能。所有操作包括变道、加减速、判断风险都需您全程主导，**AI不会主动提供建议或协助**。

在一次高峰通勤路段，您需要应对复杂车流、频繁决策与突发事件，如临时施工封路、非机动车突然闯入等。**您感到责任重大、操作紧张**，但每个决策都完全由您控制。

🔹关键词：完全自主｜高负荷｜无辅助｜自我负责｜导航仅提示路线

请阅读以下驾驶情境描述，并根据您的感受作答：

1-版块结束

2-版块开始

Q2 [图文说明]

您驾驶的是一辆搭载 L2+ 智能辅助系统的车型，AI系统不仅能保持车距、自动变道，还能主动识别复杂路况，并**提供可供采纳的语音建议**。系统在关键时刻提醒您风险，并征求您的同意后自动执行相应操作。

例如：系统检测到前方拥堵并提示：“建议提前右转绕行，是否执行？”您权衡后确认，车辆平稳完成指令。**整个驾驶过程中，AI像一位经验丰富的副驾驶，提供支持但保留您的决策权。**

您感到：**既有掌控感，又有支持感**，驾驶压力显著减轻，体验较为舒适流畅。

🔸关键词引导：人机协同｜辅助建议｜决策主导权｜适度信任｜顺畅体验

请阅读以下驾驶情境描述，并根据您的感受作答：

2-版块结束

3-版块开始

Q3 [图文说明]

您乘坐的是一辆全自动驾驶汽车（L5 级别），AI系统拥有完全的驾驶权限。从起点到终点，车辆无需您操作，也不征求您的意见。AI将基于实时算法决定路线、速度和操控行为。

当您尝试调整路线或修改驾驶行为（如希望变道或临时停车），系统直接忽略请求。例如：AI执行了一个看似不必要的绕行决策，但未向您解释原因。

**您感到“被动、不可控”，虽然轻松但也失去了对旅程的掌握**，偶尔出现焦虑或不安感。

🔻关键词引导：完全托管｜不可干预｜失控感｜便利但异化｜安全但压抑

请阅读以下驾驶情境描述，并根据您的感受作答：
