# Supplementary material for: Too much, too little, or just right? The inverted-U relationship between human-AI collaboration modes and eudaimonic well-being
Source: Front Psychol. 2026 Sep 28;17:1747252. doi: 10.3389/fpsyg.2026.1747252 (PMC13655146; doi:10.3389/fpsyg.2026.1747252)
Supplement: Supplementary file 1 [file Supplementary_file_1.zip › Experiment 1 - English.docx]

Experiment 1

Welcome to this questionnaire.

Imagine that you are embarking on an important long-distance self-drive journey from Chengdu to Xi'an (a total distance of approximately 650 kilometers, taking about 8 hours).

Section 1 begins

You are driving alone in a vehicle equipped with a traditional navigation system that provides only basic route guidance (such as traffic-light locations and turn prompts) and offers no autonomous-driving capabilities. All maneuvers—including lane changes, acceleration or deceleration, and risk assessment—must be performed entirely by you; the AI will not offer suggestions or assistance on its own initiative.

During a peak-commute stretch, you must cope with dense traffic, constant decisions, and sudden incidents—such as unannounced roadwork that blocks a lane or a non-motorized vehicle darting into your path. The responsibility feels heavy and the maneuvers stressful, yet every decision remains entirely under your control.

Keywords: Full autonomy | High workload | No assistance | Self-responsible | Navigation route-only

Please read the following driving scenario and respond based on how you would feel:

Section 1 ends

Section 2 begins

You are driving a vehicle equipped with an L2+ intelligent assistance system. The AI can maintain a safe following distance, change lanes automatically, and proactively identify complex road conditions while offering verbal suggestions you may choose to accept. At critical moments it alerts you to risks and, once you give consent, executes the corresponding maneuvers automatically.

For example: the system detects congestion ahead and prompts, “Recommend turning right early to detour—execute?” After weighing the option, you confirm, and the car completes the maneuver smoothly. Throughout the drive, the AI acts like an experienced co-pilot: supportive, yet leaving final authority in your hands.

You feel: still fully in control, yet comfortably backed up; driving stress drops noticeably, and the experience is smooth and reassuring.

Keyword cues: Human–machine collaboration | Assistive suggestions | Decision authority | Moderate trust | Smooth experience

Please read the following driving scenario and answer based on how you would feel.

Section 2 ends

Section 3 begins

You are riding in a fully autonomous vehicle (Level 5).
The AI system holds complete driving authority: from origin to destination it needs no input from you and never asks permission. Algorithms alone choose the route, set the speed, and execute every maneuver in real time.

When you try to adjust the route or change driving behavior (such as requesting a lane change or an unscheduled stop), the system simply ignores you.
For example: the AI executes a detour that seems unnecessary, yet offers no explanation.

You feel “passive and out of control”: the ride is effortless, but you have lost command of the journey, and flashes of anxiety or uneasiness surface from time to time.

Keyword guide: Full delegation | Non-intervenable | Loss of control | Convenient yet alienated | Safe yet oppressive

Please read the following driving scenario and answer based on how you would feel.

Section 3 ends
