# Supplementary material for: Too much, too little, or just right? The inverted-U relationship between human-AI collaboration modes and eudaimonic well-being
Source: Front Psychol. 2026 Sep 28;17:1747252. doi: 10.3389/fpsyg.2026.1747252 (PMC13655146; doi:10.3389/fpsyg.2026.1747252)
Supplement: Supplementary file 1 [file Supplementary_file_1.zip › Appendix A. Card-Sorting Task Instructions and Scenarios.doc]

Appendix A. Card-Sorting Task Instructions and Scenarios

Instruction to Participants

The following descriptions illustrate different ways in which humans and artificial intelligence (AI) can work together when making decisions.

Please read each description carefully and group them into several categories according to your own understanding.

There are no right or wrong answers — please categorize them in a way that feels most natural to you.

After grouping, please assign a label to each category and briefly explain why you think the scenarios within that group belong together.

**Scenarios**

1.You independently research all available options and decide what to purchase without any assistance.

2.The AI system organizes relevant data for you to review, but you make the final decision yourself.

3.The AI provides several options and explains their advantages and disadvantages, and you choose the one you prefer most.

4.The AI recommends a particular action but requires your consent before proceeding.

5.The AI directly completes the decision for you, but you can command it to redo the process if you are dissatisfied.

6.The AI makes and executes the decision entirely on its own, without accepting any modification or feedback.
